# Correction: Evidence for adaptive evolution in the receptor-binding domain of seasonal coronaviruses OC43 and 229e

**DOI:** 10.7554/eLife.83277

**Published:** 2022-09-14

**Authors:** Kathryn E Kistler, Trevor Bedford

**Keywords:** Viruses

Kistler KE, Bedford T. 2021. Evidence for adaptive evolution in the receptor-binding domain of seasonal coronaviruses OC43 and 229e. *eLife* **10**:e64509. doi: 10.7554/eLife.64509.

Published 19 January 2021

We noticed that the rates of adaptation we reported were given in the units of adaptive mutations *per nucleotide* per year, however the y-axis labels and text state that the units are adaptive mutations *per codon* per year. Thus, the rates we published were 3 times lower than they should be. We have updated all figures and text to give rates per codon. This change affects the rates and the absolute number of adaptive mutations that were reported in the original manuscript, but the relationship between viruses and the conclusions about adaptive evolution do not change.

The corrected Figure 4 is shown here:

**Figure 1:**
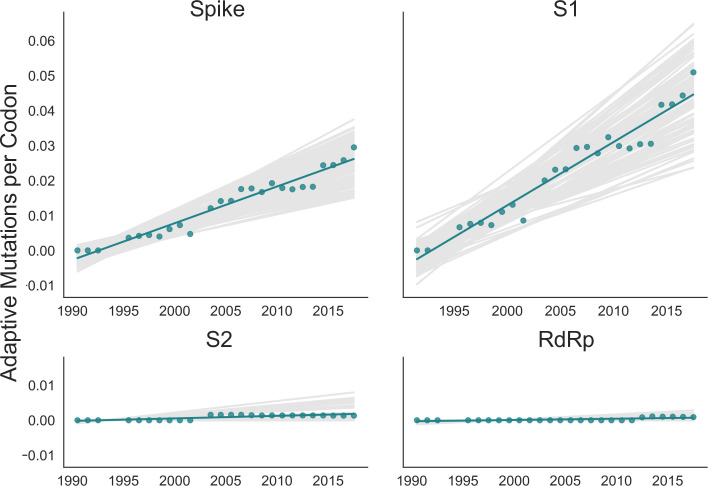


The originally published Figure 4 is shown here for reference:

**Figure 2:**
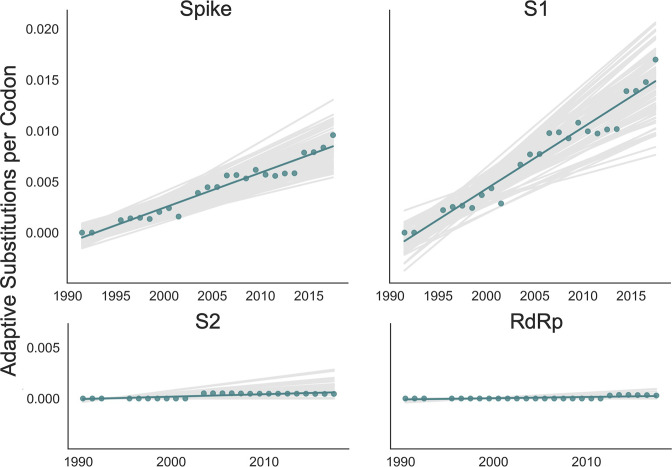


We also noticed that the Figure 4 legend states that the points in the plot are red when they are, in fact, green. We have altered the legend for Figure 4 to correct this and have underlined these changes below. Corrected Figure 4 legend:

Green dots display estimated values calculated from the empirical data and green lines show linear regression fit to these points.

Original, published legend:

Red dots display estimated values calculated from the empirical data and red lines show linear regression fit to these points.

The corrected Figure 5 is shown here:

**Figure 3:**
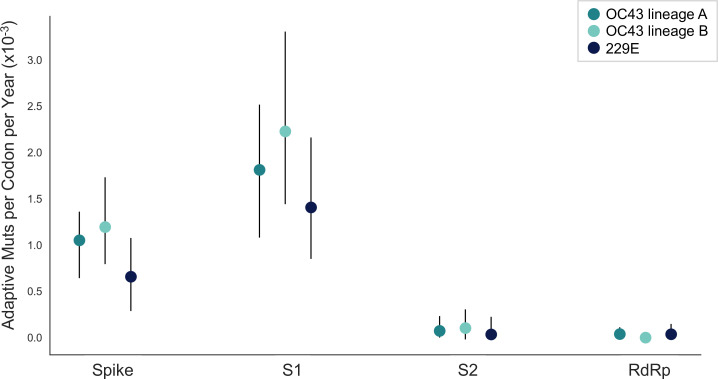


The originally published Figure 5 is shown here for reference:

**Figure 4:**
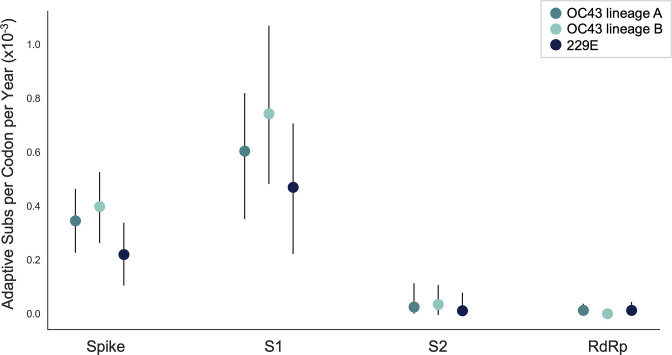


The corrected Figure 5, Supplement 1 is shown here:

**Figure 5:**
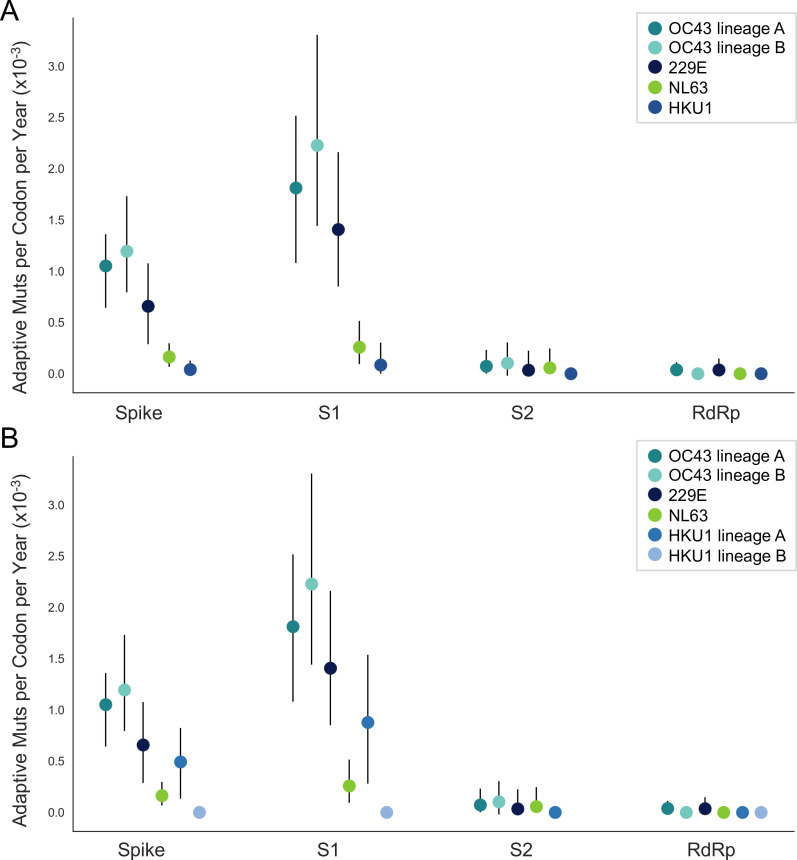


The originally published Figure 5, Supplement 1 is shown here for reference:

**Figure 6:**
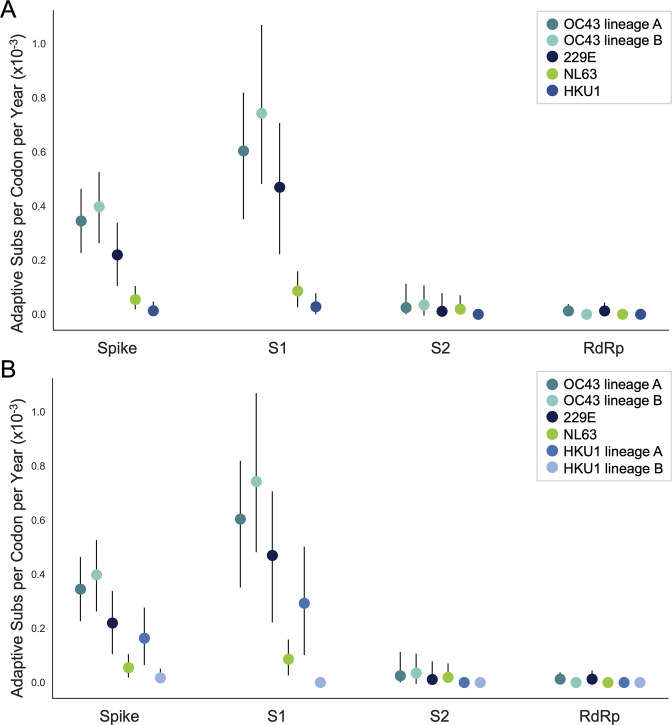


The corrected Figure 6 is shown here:

**Figure 7:**
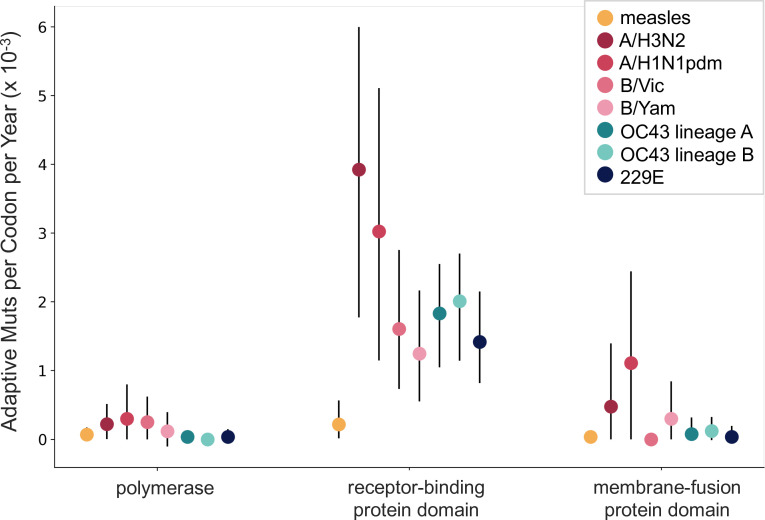


The originally published Figure 6 is shown here for reference:

**Figure 8:**
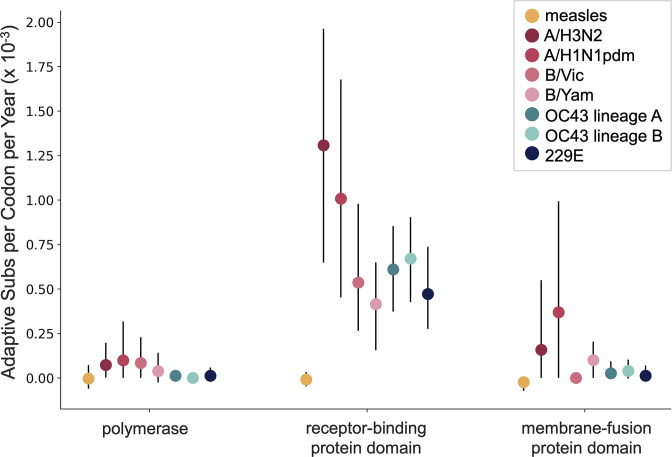


The corrected Figure 7 is shown here:

**Figure 9:**
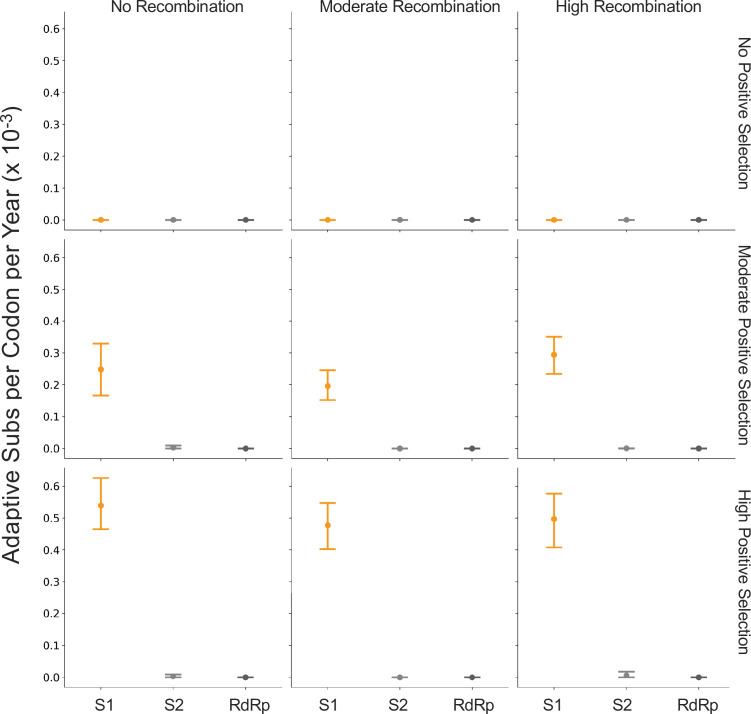


The originally published Figure 7 is shown here for reference:

**Figure 10:**
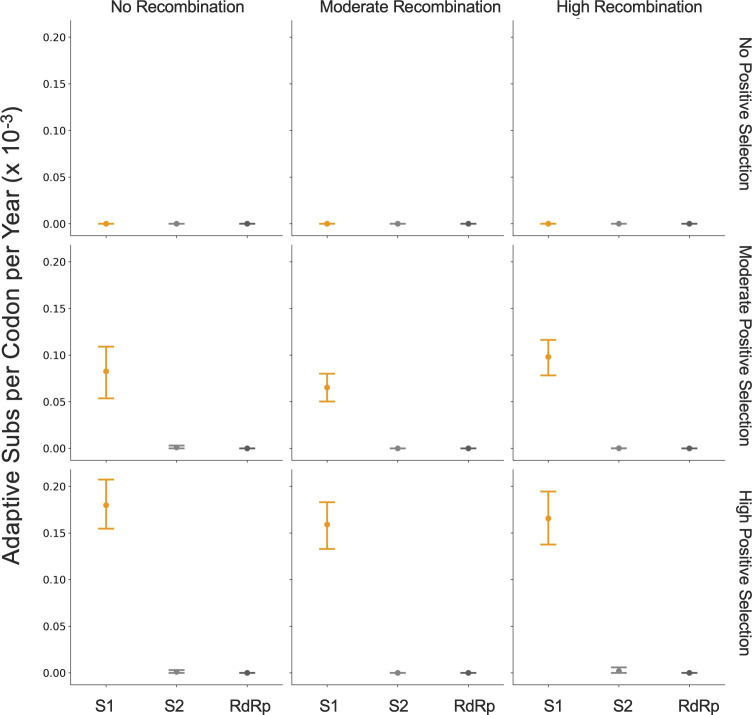


The corrected Figure 7, Supplement 1 is shown here:

**Figure 11:**
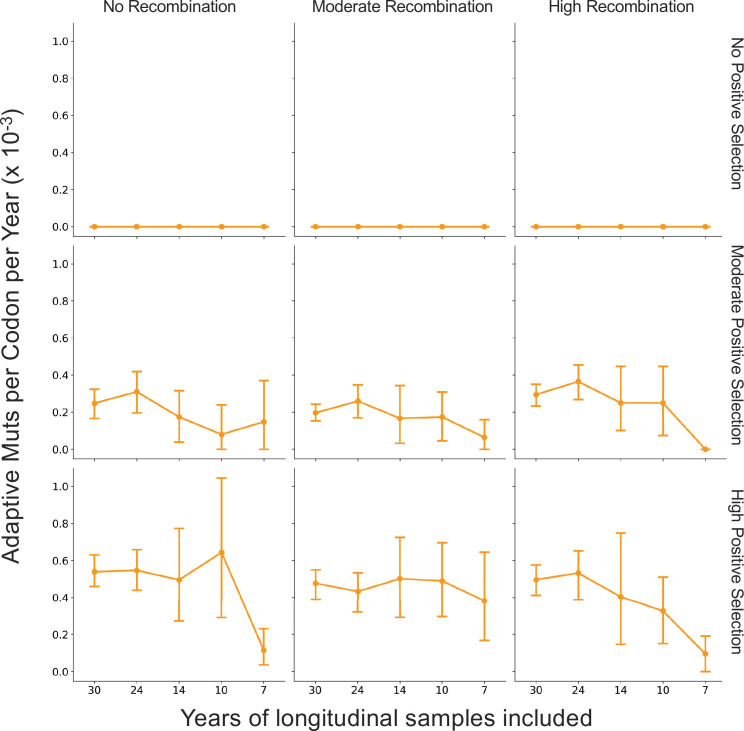


The originally published Figure 7, Supplement 1 is shown here for reference:

**Figure 12:**
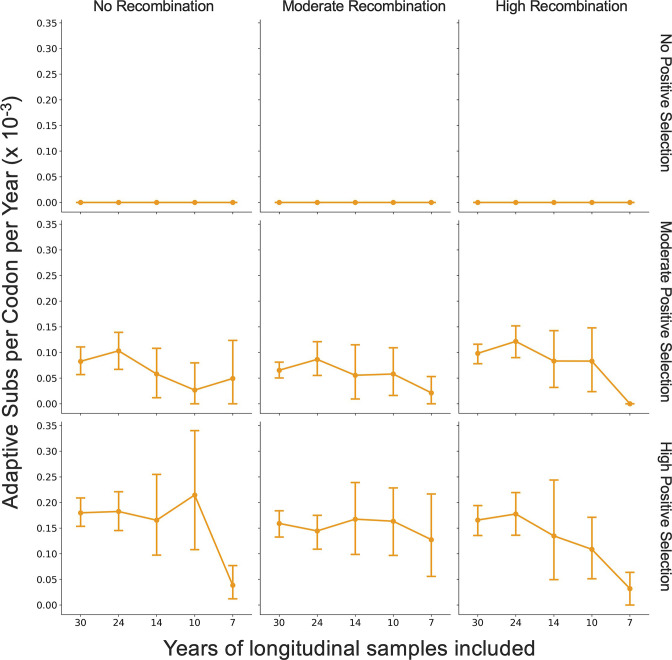


The rates are mentioned once in the Results section and once in the Discussion. The sentences mentioning these rates have been corrected to give the rates in adaptive mutations per codon, and the changes have been underlined below. Corrected sentences in the Results section:

We estimate that OC43 lineage A accumulates roughly 1.8×10^–3^ adaptive substitutions per codon per year (or 1.4 adaptive amino acid substitutions in S1 each year) in the S1 domain of spike, while the rate of adaptation in OC43 lineage B is slightly higher and is estimated to result in an average 1.7 adaptive substitutions in S1 per year (Figure 5). The S1 domain of 229E is estimated to accrue 0.76 adaptive substitutions per year (a rate of 1.4×10^–3^ adaptive substitutions per codon per year).

Sentences from the original, published manuscript:

We estimate that OC43 lineage A accumulates roughly 0.61×10^–3^ adaptive substitutions per codon per year (or 0.45 adaptive amino acid substitutions in S1 each year) in the S1 domain of spike, while the rate of adaptation in OC43 lineage B is slightly higher and is estimated to result in an average 0.56 adaptive substitutions in S1 per year (Figure 5). The S1 domain of 229E is estimated to accrue 0.26 adaptive substitutions per year (a rate of 0.47×10^–3^ adaptive substitutions per codon per year).

Corrected sentence in the Discussion section:

We observe that S1 accumulates between 0.8 (229E) and 1.4 (OC43) adaptive substitutions per year.

Sentence in the original, published manuscript:

We observe that S1 accumulates between 0.3 (229E) and 0.5 (OC43) adaptive substitutions per year.

Additionally, we noticed that our original manuscript states that the measles polymerase gene is P, but the polymerase (and the gene we calculated rates of adaptation for) is actually called L. We also noticed a typo, where the HCoV polymerase is referred to as RbRd, instead of RdRp. We have corrected this in the legend for Figure 6 and in the Methods section and underlined the changes below.

The corrected sentence in the legend for Figure 6:

For measles, the polymerase is the L gene, the receptor-binding protein is the H gene, and the fusion protein is the F gene.

The original sentence in the Figure 6 legend, for reference:

For measles, the polymerase is the P gene, the receptor-binding protein is the H gene, and the fusion protein is the F gene.

The corrected sentence in the Methods:

The polymerase for influenza was PB1, for measles was the L protein, and for the HCoVs was RdRp (nsp12).

The original sentence in the Methods, for reference:

The polymerase for influenza was PB1, for measles was the P protein, and for the HCoVs was RbRd (nsp12).

